# Taste Compounds and Polyphenolic Profile of Tomato Varieties Cultivated with Beneficial Microorganisms: A Chemical Investigation on Nutritional Properties and Sensory Qualities

**DOI:** 10.3390/biom13010117

**Published:** 2023-01-06

**Authors:** Carlo Francesco Morelli, Adele Cutignano, Giovanna Speranza, Gennaro Roberto Abbamondi, Marco Rabuffetti, Carmine Iodice, Rocco De Prisco, Giuseppina Tommonaro

**Affiliations:** 1 Department of Chemistry, University of Milan, Via Golgi 19, 20133 Milan, Italy; 2 National Research Council (CNR), Institute of Biomolecular Chemistry (ICB), Via Campi Flegrei, 34, 80078 Pozzuoli, Italy

**Keywords:** tomato, effective microorganisms (EM), polyphenols, umami-taste compounds, glutamic acid, aspartic acid, AMP, GMP, LC–MS, NMR

## Abstract

There is a strong need to develop eco-sustainable agricultural techniques to improve crop yields while preserving biomolecule contents and reducing the adverse environmental impact of agro-chemicals. The use of microorganisms in agriculture represents an attractive and innovative solution. Herein, a chemical study on the nutritional and sensory qualities of San Marzano Cirio 3 (SMC3), Corbarino (CO) and Brandywine (BW) tomato varieties cultivated with and without effective microorganisms (EM) is reported. LC–MS analysis of the methanolic extracts allowed for the identification of 21 polyphenol derivatives. In different proportions among the studied varieties, the two main polyphenols were rutin and naringenin chalcone; the latter was isolated and chemically identified by complementary HR-ESIMS/MS and NMR methods. SMC3 and CO were richer in naringenin chalcone. Conversely, BW showed higher proportions of rutin; however, in all cases, the relative amounts of the two polyphenols considered together increased over the other minor components after the EM treatment. The qualitative and quantitative HPLC analyses of taste-active compounds (aspartic acid, glutamic acid, AMP and GMP) revealed a significant difference in aspartic and glutamic acids and ribonucleotide contents according to the cultivation condition (±EM), particularly in BW. This study provides chemical data in support of the use of EM green technology for the cultivation of edible agricultural products, such as tomato preserves, and may even improve nutritional and sensory qualities while safeguarding the environment.

## 1. Introduction

Tomatoes and tomato-based products are one of the most representative foods of the Mediterranean diet and are consumed worldwide. The mode of consumption of this food is varied: it can be used whole, cut, raw or cooked. Over the years, tomatoes have acquired considerable importance in the food sector due to consumer awareness of their good nutritional properties and sensory qualities [1,2]. The main contributors to the healthy properties of tomatoes are lycopene and β-carotene, two carotenoids that are important nutraceutical constituents of the human diet for their antioxidant activity [3,4,5]. Besides carotenoids, tomatoes are a rich source of polyphenols, whose beneficial effects in the prevention and treatment of several human diseases have been demonstrated in many epidemiological studies [6,7].

The content of these metabolites depends on the genetic and environmental conditions, agricultural practices and grade of ripening [8,9,10,11,12]. As some secondary compounds are relevant for color, flavor or tissue specificities, these changes in fruit development are closely related to the metabolite content of tomato fruits. Agricultural practices could also affect the biosynthesis of secondary metabolites. The search for “green” cultivation strategies through reductions in chemical fertilizers and pesticides is a topic of growing interest. One of the most promising strategies for agricultural sustainability is the use of plant growth-promoting bacteria (PGPB). Plant-associated microorganisms can improve plant growth and health through different processes [13,14,15]. Effective microorganisms (EM) are a pool of beneficial microorganisms containing more than 80 species (photosynthetic bacteria, lactic acid bacteria, yeasts, actinomycetes, etc.) isolated from the soil by Higa et al. [16]. Previous studies have discussed the positive effects of EM technology in terms of crop production, fruit qualities and resistance to pathogens [17,18,19]. A recent study suggested that the application of EM technology could improve the content of bioactive compounds in tomato fruits, in particular polyphenols with renowned antioxidant activity [8]. Among the secondary metabolites, phenols and flavonoids constitute important nutraceutical components of the human diet and phytochemicals that modulate plant resistance to stress conditions and influence fruit postharvest performance and shelf life [20].

On the other hand, volatile aromatic compounds, sugars, organic acids, free amino acids and salts are responsible for the flavor and taste of tomatoes [2]. The glutamic acid/aspartic acid ratio seems to be the key factor for tomato taste. It should also be noted that the amount of free amino acids present increases up to a maximum of five times during maturation. The reason for this increase is found in the proteases of the tomato, which, by their actions, allow for the degradation of polypeptides containing glutamate as a terminal amino acid and the subsequent release of free amino acids, including L-glutamic acid [21]. In addition to protein degradation, during the maturation process, we witnessed the degradation of nucleic acids, resulting in an increase in the concentration of 5′-purine ribonucleotides, such as AMP, GMP and IMP, derived from AMP [22]. For this reason, the riper a tomato is, the greater the taste perceived by the consumer.

Two typical tomato varieties of the Campania region (Southern Italy), namely San Marzano (SM) and Corbarino (CO), were selected for our study. In particular, “San Marzano Cirio 3” (SMC3) is a variety selected by farmers over the years with the aim to improve its cultivation parameters (increased plant productivity and better resistance to diseases). It is known for its nutritional features and global commercial importance, especially in processing products (peeled, pureed, etc.). Corbarino, a typical variety of the Corbara area, has also shown interesting nutritional properties [3,4]. Brandywine (BW) is a tomato variety, which is typical for North America. It was chosen as a foreign tomato variety to be compared with the typical Italian varieties.

This study aims to contribute to the investigation of the flavor and health-related compound features of tomatoes and to discuss how different cultivation techniques can affect sensory and functional factors.

## 2. Materials and Methods

### 2.1. General

NMR spectra were recorded in CD_3_OD on a 600 MHz Bruker Avance III spectrometer (Bruker BioSpin GmbH, Rheinstetten, Germany) equipped with a CryoProbeTM at 600.15 MHz. The chemical shift values were reported in ppm (δ) and referenced to solvent residual protons (CD_3_OD ^1^H δ 3.34, ^13^C 49.0 ppm). High-resolution mass spectra were acquired on a QExactive hybrid quadrupole-orbitrap mass spectrometer (Thermo Scientific, Milan, Italy) on-line with the UHPLC apparatus Infinity 1290 (Agilent Technologies, Santa Clara, CA, USA). HPLC analyses were performed on a Shimadzu System LC 6A with a UV–VIS detector SPD 10A VP, a CR 3A recorder and a system controller SCL 10A VP (polyphenols) or on a LC-4000 (Jasco) instrument connected to a UV/Vis detector model UV 4070 (Jasco) and interfaced to a PC running the ChromNav software package (Chromatography Data System, Jasco) and Chemstation integration software Class–VP 5.0 (glutamic and aspartic acids, AMP and GMP). The chemical standards and solvents (HPLC/MS-grade) were purchased from Merck (Milan, Italy).

### 2.2. Tomato Cultivation and Harvesting

An experimental field covering approximately 1000 square meters was dedicated to the growth of the tomato plant samples. The field was located in Nocera Inferiore, Italy, under conditions of 11.3 h of daylight and 70% relative humidity. The plants (50 of each variety and treatment) were divided into two groups based on the fertilizers used during growth: traditional fertilizers (composted solid manure containing approximately 9% N–P–K–Mg minerals, 38% organic carbon and 11% humic acid) and effective microorganisms (EM) [8]. A total of 50 tomato samples of each variety and treatment were randomly selected and harvested at their peak of ripeness and then stored at −20 °C in the laboratory until analysis.

### 2.3. Sample Preparation

For the taste component evaluation, four fruits of SMC3 (±EM) and BW (±EM) (220–250 g) and ten fruits of CO (±EM) (180–200 g) were divided into three parts, which included the peel, the outer flesh and the inner jelly pulp, and were considered separately for each tomato variety and treatment. The three parts of the fruits were divided, freeze-dried and conserved at −18 °C until analysis. The amounts of compounds were expressed as percentages with respect to the lyophilized material.

For the assessment of the polyphenolic profile, four fruits of SMC3 (±EM) and BW (±EM) (100–180 g) and eight fruits of CO (±EM) (120–130 g) were analyzed. Frozen tomato samples were homogenized in a blender and successively extracted with methanol (1:2 *w*/*v*; 30 min under shaking in the dark; repeated 3 times with fresh solvent). The sample weights and corresponding yields of the extracts are reported in Table S1 (Supplementary Materials).

### 2.4. Polyphenols

#### 2.4.1. LC–MS/MS Analysis of Polyphenols

The polyphenol analysis of tomato extracts (2 mg/mL) was achieved by UHPLC on a Kinetex core–shell C18 column (75 mm × 2.1 mm, 100 A, 2.6 µm) (Phenomenex, Castel Maggiore (BO), Italy) coupled to a QExactive mass spectrometer equipped with a HESI source operating in negative ion mode, according to a previously reported methodology [23]. The LC–MS data were processed by the Xcalibur software (version 3.0.63, Thermo Scientific, San Jose, CA, USA).

#### 2.4.2. Isolation of Naringenin Chalcone

An aliquot of the methanolic extract of SMC3 was fractionated by semi-preparative HPLC on a Luna C18(2) column 250 × 10 mm, 5μm (Phenomenex) by using the following eluents: A, water + 0.3% trifluoroacetic acid (TFA), and B, acetonitrile (ACN). The following elution gradient was used: 60:40 A/B to 100% B, *v*/*v*, linear gradient 0–25 min, 40% to 50% B; 25–60 min, 50% to 100% B and return to the starting condition in 10 min; flow rate, 4 mL min^−1^; the run time was 60 min; UV detector λ **=** 210 and 260 nm; the sensitivity was adjusted to 0.04 AUFS; room temperature. The major peak 16, eluting at 50 min, afforded 0.6 mg of a pure compound whose identification as naringenin chalcone was ascertained by LC-HRESIMS/MS and NMR analysis in CD_3_OD (Supplementary Materials, Figures S1–S4 and Table S2).

### 2.5. Taste Components

#### 2.5.1. Extraction of Glutamic and Aspartic Acids

A FlashVac Biotage system was used for the filtration in the sample preparation, which employed 25 mL Isolute tubes and 20 μm porous septa.

An extraction and a pre-column derivatization procedure were carried out as previously described [24]. Briefly, a lyophilized tomato sample (100 mg) was extracted four times with 0.1 M sodium borate buffer at pH 8.5 (4 mL) at 21 °C for 30 min under stirring conditions. After the filtration, equal amounts of each extraction were combined and used for analysis.

A mixture of sample solution (250 μL), 5 mM S-methylcysteine as the internal standard (100 μL), 0.1 M sodium borate buffer pH 8.5 (250 μL) and 1-fluoro-2,4-dinitrobenzene (Sanger’s reagent) solution in acetone (5 mM, 500 μL) was heated at 70 °C for 45 min in a Pyrex tube equipped with a perforated screw cap and a septum. A needle was then inserted through the septum, and most of the acetone was evaporated by continuous heating for an additional 10 min. The dilution of the derivatized solution with an equal amount of 0.1% aqueous TFA afforded the sample for HPLC analysis. A comparison of the retention times and co-elution with authentic samples of 2,4-dinitrophenyl derivatives of glutamic and aspartic acids allowed for the identification of the glutamic and aspartic acid peaks, respectively, in the chromatograms. Calibration curves were obtained for quantitative analyses from the solutions of glutamic and aspartic acids at increasing concentrations following the same derivatization method.

#### 2.5.2. HPLC Quantitative Analysis of Glutamic and Aspartic Acids

An RP-18 Gemini column (250 mm × 4.6 mm i.d., 5 µm, Phenomenex) was used for the analysis of the amino acids, and the following linear gradient of 0.1% aqueous TFA (solvent A) and ACN/solvent A 80:20 (solvent B) was used: 0–10 min, isocratic elution solvent A: solvent B 80:20; 10–15 min, gradient to solvent A: solvent B 70:30; 15–25 min, gradient to solvent A: solvent B 60:40; 25–30 min, isocratic elution solvent A: solvent B 60:40; 30–35 min, gradient to solvent A: solvent B 40:60; 35–45 min, isocratic elution solvent A: solvent B 40:60; 40–60 min, gradient to solvent A: solvent B 80:20. The flow rate was 0.75 mL/min; the detection was set at λ = 356 nm; the injection volume was 20 µL. Millex-HV hydrophilic PVDF filters of 0.45 um (Millipore) were used for ultrafiltration of the samples before the HPLC analysis.

For the calibration curves, starting from a 50 mM solution, 5 and 2.5 mM solutions of the analytes were obtained by successful dilutions. The samples of the diluted solutions were derivatized as described and analyzed by HPLC as in Section 2.5.2. The calibration curves for glutamic and aspartic acids were constructed by plotting the ratio of the peak area of the analyte divided by the peak area of the internal standard against the concentrations of the starting solutions and considering the zero point (Supplementary Materials, Figure S5).

#### 2.5.3. Extraction of AMP and GMP

The procedure described in [25] was applied with some modifications [24]. A FlashVac Biotage system was used for the filtration in the sample preparation, which employed 25 mL Isolute tubes and 20 µm porous septa. A lyophilized tomato sample (500 mg) was extracted three times in a fritted tube with 0.05 M HCl (10 mL) for 30 min at 21 °C. After the filtration, equal amounts of each filtrate were combined and filtered through a 0.45 μm Millipore filter, after which they were analyzed by HPLC using the method reported in Section 2.5.4.

#### 2.5.4. HPLC Quantitative Analysis of AMP and GMP

A LiChrospher 100 RP-18 (5 µm) LiChroCART column (250 mm × 4.6 mm i.d.) was used for the ribonucleotide analysis. The elution was performed in isocratic mode using aqueous TFA 0.1% and ACN at a 99.5:0.5 ratio. The flow rate was 0.750 mL/min; the detection was set at λ = 254 nm; the injection volume was 20 µL.

GMP and AMP solutions at increasing concentrations (0, 3.125, 6.250 and 12.500 mg/100 mL) were used for the calibration. The calibration curves were obtained by plotting the concentrations of the solutions against the peak areas in the HPLC chromatograms (Supplementary Materials, Figure S6).

### 2.6. Statistical Analysis

To determine significant differences in the concentrations of glutamic acid, aspartic acid, AMP and GMP within the three parts of the fruits and among the different varieties considered, we used statistical analysis techniques, which included an analysis of variance (ANOVA) and a Tukey’s post-hoc test at a 95% confidence level. These calculations were performed using the PSPP software, which is available at www.gnu.org/software/pspp.

The extraction of the analytes, the derivatization procedure (when requested) and the analyses were carried out in triplicate.

## 3. Results and Discussion

### 3.1. Identification of Polyphenols in SMC3, CO and BW Varieties of Tomato

The LC–ESI-MS analyses of the methanolic extracts showed similar qualitative profiles, yet differences were appreciable among the three varieties and within each variety between the traditional and EM cultivation approaches. Overall, 21 polyphenol derivatives were identified. Seven of these compounds, namely chlorogenic acid, caffeic acid, p-coumaric acid, rutin, quercetin-3-*O*-glucoside, eriodictyol and naringenin, were identified by comparison with authentic standards run in the same chromatographic conditions. Naringenin chalcone was isolated and unambiguously chemically characterized by complementary HR-ESIMS/MS and NMR methods (see Materials and Methods and Supplementary Materials, Figures S1–S4, Table S2); the remaining metabolites were putatively identified by accurate mass, interpretation of MS/MS spectra and comparison with experimental data reported in the literature [26,27,28] (Table 1).

In particular, minor dicaffeoyl-quinic acid isomers I-IV (C_25_H_24_O_12_) were putatively identified by high-resolution mass measurements and interpretation of the fragmentation pattern of a molecular ion at [M-H]^−^ *m/z* 515.1178 (Supplementary Materials, Figure S7). In fact, all of these isomers gave diagnostic fragment ions at *m*/*z* 353.0892/335.0761 (monocaffeoyl-quinic acid/-H_2_O), 191.0556 (quinic acid) and 179.0343 (caffeic acid). Analogously, the deprotonated ion at *m/z* 677.1501 was assigned to a tricaffeoyl-quinic acid derivative since it exhibited an MS^2^ fragmentation pattern at *m/z* 515.1201, 353.0886, 335.0779, 191.0556 and 179.0343, clearly overlapping with those registered for the dicaffeoyl isomers (Supplementary Materials, Figure S8). The caffeic acid hexoside isomers I-III at *m/z* 341.0868 were assigned based on the observed ion at *m/z* 179.0343 in the MS/MS spectra corresponding to caffeic acid, arising from the neutral loss of a hexose moiety (M-162) (Supplementary Materials, Figure S9). In addition, they eluted before the corresponding aglycone (t_R_ 0.55, 0.61 and 0.79 vs. 1.02 min for caffeic acid), as expected [27,28]. Along with rutin (quercetin-3-*O*-rutinoside) and quercetin-3-*O*-glucoside, which were confirmed by authentic standards, another quercetin glycoside was putatively identified, i.e., rutin pentoside at *m/z* 741.1904 (MS^2^ = *m/z* 300.0279), which is already reported in tomatoes [26,29].

Eriodictyol co-occurred with a possible isomer and a methoxy derivative. The latter was inferred from an accurate mass measurement of the ion at *m/z* 301.0707, which was attributed to C_16_H_13_O_6_ ([M-H]^−^) and putatively listed as hesperetin. The high resolution allowed us to distinguish it from quercetin (C_15_H_10_O_7_ accounts for [M-H]^−^ *m/z* 301.0353), which is also found in the literature as a tomato component [7,26,30], but it was not revealed in our analysis even at a different retention time.

The compound at *m/z* 593.1501 gave a product ion at *m/z* 285.0396 and was identified as kaempferol rutinoside, which has previously been reported in tomatoes [26].

### 3.2. Composition Analysis of Polyphenols in SMC3, CO and BW Varieties of Tomato

Table 1 reports the relative amounts of the various polyphenols in the three tomato varieties studied, grown with and without EM technology.

As shown, all of the investigated varieties contained the flavonol glycoside rutin and the flavonoid intermediate naringenin chalcone as the main polyphenolic components in different proportions. Other glycosides accounted for less than 5% each (caffeic acid hexosides, rutin pentoside, quercetin hexoside, kaempferol rutinoside, naringenin hexoside). Minor components were represented by caffeic acid derivatives (chlorogenic acid and mono, di and tricaffeoyl-quinic acid derivatives) and the flavonoids eriodictyol, hesperetin and naringenin. Phenolic acids, i.e., caffeic and p-coumaric acids, also occurred in low amounts.

Naringenin chalcone is the main polyphenol in tomato peel, and its anti-inflammatory and anti-allergic properties have been proven in *in vivo* tests [31,32]. In addition, chalcones are ranked highly for their ability to inhibit human liver cancer cell growth *in vitro* with respect to other polyphenol derivatives [33].

On the other hand, the multiple beneficial properties of rutin, also called vitamin P, have been extensively reported, including neuroprotective, anti-inflammatory, anti-proliferative, antidiabetic and chemotherapeutic effects [34]. Very recently, its use as a bioconjugate has been explored in *in vitro* and *in ovo* tests [35].

Both cultivars from the Campania region, SMC3 and CO, were richer in naringenin chalcone. The EM technology approach seemed to have led to an enhancement of the biosynthetic pathway towards naringenin chalcone as the end product, while rutin was less represented in the pool, although still being one of the two most abundant polyphenols. Conversely, in BW, in which the relative amounts of the two metabolites are reverted, rutin accumulates mainly under EM cultivation. However, when we considered the sum of the relative contributions of rutin and naringenin calchone over the total amount of polyphenols in the three tomato varieties, the EM technology led to an increase in the two bioactive components, converging the biosynthesis toward the accumulation of these two main derivatives.

### 3.3. Influence of EM Technology on the Umami-Tasting Components of Tomatoes

The palatability and acceptance of foods are related to the presence of taste-active compounds. Among these, naturally occurring umami-tasting molecules are of special interest due to their taste-enhancing action and their mutual synergistic effect.

It has been recognized that free amino acids, such as glutamate and aspartate, and the ribonucleotides adenosine monophosphate (AMP) and guanosine monophosphate (GMP) are responsible for the umami taste of tomato [25]. Thus, the content of these taste-active compounds was assessed in fruits cultivated using the EM technology and compared to that of fruits cultivated using the traditional method.

Glutamate and aspartate were extracted with borate buffer at pH 8.5, following a previously described protocol [24]. Quantitative HPLC analyses were carried out using the pre-column derivatization method with 1-fluoro-2,4-dinitrobenzene (Sanger’s reagent) and S-methyl-cysteine as the internal standard. Authentic samples of glutamic and aspartic acids at different concentrations were used for peak attribution and the construction of calibration curves for quantitative measurements.

**Figure 1:**
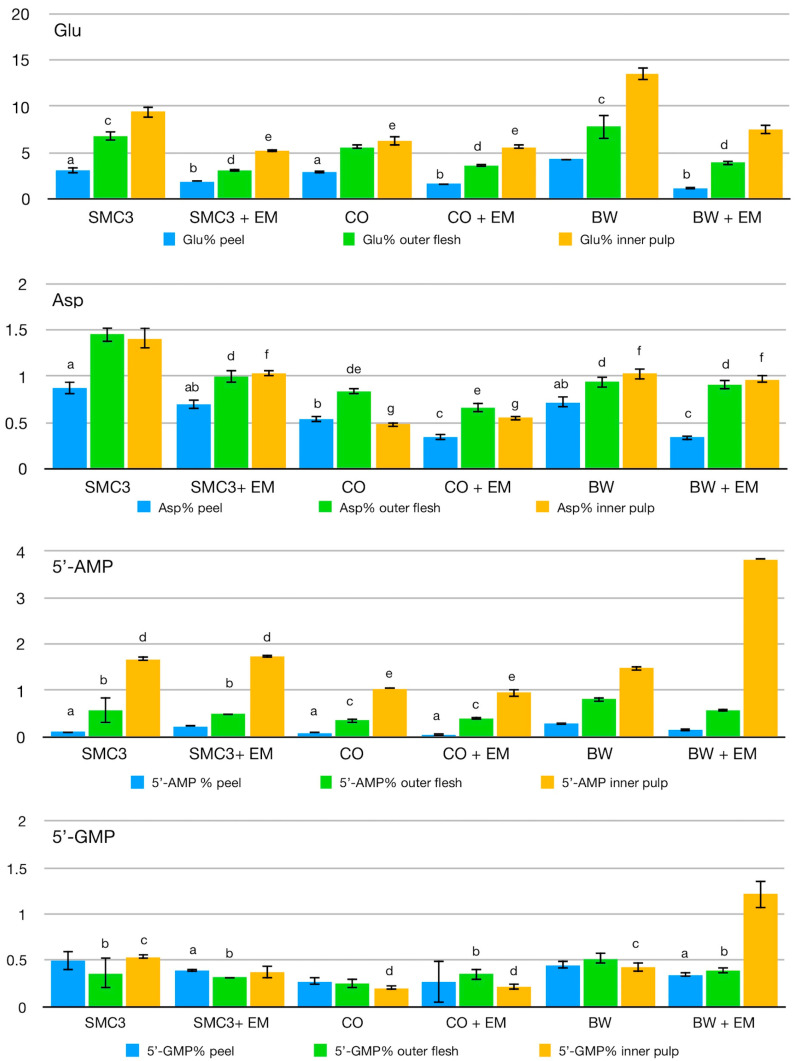
Glutamic acid, aspartic acid, AMP and GMP contents in tomatoes cultivated using standard methods and EM technology. The average values of the three independent measurements are reported; bars marked with the same letter did not show statistically relevant differences at a 95% confidence level after ANOVA analysis and Tukey post-hoc test. SMC3: San Marzano Cirio 3; SMC3 + EM: San Marzano Cirio 3 cultivated using EM technology; CO: Corbarino; CO + EM: Corbarino cultivated using EM technology; BW: Brandywine; BW + EM: Brandywine cultivated using EM technology.

Although the glutamic acid content in the three parts of the fruit can vary widely, higher concentrations are usually found in the outer flesh and in the inner pulp [24,25]. In the varieties considered, the distribution of glutamic acid in the three parts of the fruit follows a common trend: it is at a minimum in the peel, becomes higher in the outer flesh and reaches a maximum in the inner pulp. A higher concentration of glutamic acid was found in the inner pulp of the BW variety, followed by SMC3 and CO (the inner pulp). In the outer flesh of SMC3 and BW, the glutamic acid content did not differ significantly, while CO showed the lowest content. From the graph in Figure 1, it appears that the application of EM technology is detrimental to the glutamic acid content. The amount of glutamic acid in the three parts of the fruits cultivated with the application of EM technology was lower than that measured in the fruits cultivated using the traditional method. Only the glutamic acid content in the inner pulp of CO + EM was comparable to that measured in the fruits cultivated using the traditional method.

Aspartic acid appears to be distributed differently from glutamic acid among the three parts of the fruits. SMC3 showed the highest content of aspartic acid in the outer flesh and in the inner pulp without statistically relevant differences. The application of EM technology lowered the content of aspartic acid, but did not change its equal distribution between the two parts of the fruit.

In BW, the amount of aspartic acid in the outer flesh and in the inner pulp was very similar and was not affected by the application of EM technology. A similar situation was noticed for CO, although the differences in aspartic acid content of the outer flesh for the fruits cultivated using the standard method and EM technology are almost at the limit of statistical relevance. The concentrations of aspartic acid found in the outer flesh and in the inner part of SMC3 after the EM cultivation were very similar to those found in BW and BW + EM.

The ribonucleotides AMP and GMP were extracted with 0.05 M HCl, starting from 500 mg of freeze-dried material. After the extraction, the filtered solutions were subjected to HPLC analyses. Figure 1 shows that AMP is mainly present in the inner pulp. Very low amounts of AMP were detected in the peel, and an intermediate concentration was found in the outer flesh. SMC3 showed the highest content of AMP in the inner pulp, followed by BW and CO. The AMP content in the outer flesh was similar, although statistically different, for the three considered varieties. The application of EM technology did not affect the concentration of AMP in the outer flesh and in the inner pulp of SMC3 and CO. Conversely, BW showed a slight decrease in the outer flesh and a marked increase in the inner pulp of AMP following the application of EM technology.

The concentration of GMP was quite low in all of the analyzed fruit parts and appeared to be more evenly distributed. In CO and BW, the amounts of GMP in the peel and outer flesh were very similar, while they were slightly lower in the inner pulp. In fruits cultivated following the application of EM technology, the amount of GMP decreased in all parts of the fruit for SMC3. In CO, it increased slightly but significantly in the outer flesh, while it remained fairly constant in the inner pulp. The most evident difference after the EM treatment was noticed in BW. While the GMP content decreased in the peel and outer flesh, a three-fold increase in the inner pulp was recorded for BW + EM.

## 4. Conclusions

The present work aims to provide a chemical analysis of different varieties of tomato cultivated with the traditional method compared with those cultivated by using a pool of beneficial microorganisms (EM), which represents one of the most promising strategies in agricultural sustainability. The results pointed out the influence of EM technology on the polyphenolic profile and umami-taste compound content. In particular, our data contribute to the evidence on the safe and useful application of microorganisms as agricultural practices for cultivating edible products by preserving and, in some cases, improving nutritional and sensory qualities while safeguarding the environment.

## Figures and Tables

**Table 1:** Polyphenolic identification in tomato extracts by UHPLC–ESIMS^−^ and relative composition (%) based on peak area integration of the extracted molecular ions.

Compound	t_R_ (min)	Measured *m/z* [M-H]^−^	Molecular Ion Formula	Calculated *m/z* [M-H]^−^	MS^2^	SMC3	SMC3 + EM	CO	CO + EM	BW	BW + EM
Caffeic acid hexoside I	0.55	341.0868	C_15_H_17_O_9_^−^	341.0878	179,135	1.7	0.8	4.4	2.7	6.3	1.6
Caffeic acid hexoside II	0.61	341.0868	C_15_H_17_O_9_^−^	341.0878	179,135	2.3	1.2	4.9	2.6	6.0	2.0
Caffeic acid hexoside III	0.79	341.0868	C_15_H_17_O_9_^−^	341.0878	179,135	3.4	1.8	7.2	4.0	5.3	2.4
Chlorogenic acid *	0.86	353.0869	C_16_H_17_O_9_^−^	353.0878	191, 173, 135	3.7	2.6	11.5	8.2	8.8	2.5
Caffeic acid *	1.02	179.0335	C_9_H_7_O_4_^−^	179.0344	135	1.0	0.4	1.1	0.6	1.8	0.6
Rutin pentoside	1.39	741.1904	C_32_H_37_O_20_^−^	741.1884	300	4.8	3.1	6.5	3.7	4.4	2.3
p-Coumaric acid *	1.41	163.0385	C_9_H_7_O_3_^−^	163.0395	119	0.1	0.1	0.0	0.0	0.4	0.1
Rutin *	1.53	609.1457	C_27_H_29_O_16_^−^	609.1461	300	25.1	20.5	25.1	21.0	43.6	56.4
Quercetin-3-*O*-glucoside *	1.65	463.0874	C_21_H_19_O_12_^−^	463.0882	300	0.1	0.0	0.1	0.1	0.1	0.6
Dicaffeoyl-quinic acid I	1.75	515.1184	C_25_H_23_O_12_^−^	515.1195	353, 173, 179	0.6	0.5	2.1	1.5	0.6	0.3
Kaempferol-rutinoside	1.77	593.1501	C_27_H_29_O_15_^−^	593.1512	285	1.0	0.7	0.6	0.5	0.9	2.1
Dicaffeoyl-quinic acid II	1.88	515.1184	C_25_H_23_O_12_^−^	515.1195	353, 191, 179	0.8	0.3	1.4	1.3	0.7	0.3
Dicaffeoyl-quinic acid III	2.10	515.1184	C_25_H_23_O_12_^−^	515.1195	353, 173, 179	0.8	1.0	5.9	7.8	2.5	0.7
Naringenin hexoside	2.33	433.1130	C_21_H_21_O_10_^−^	433.1140	271, 151, 119	3.2	1.4	1.4	1.1	1.1	1.8
Eriodictyol *	2.70	287.0568	C_15_H_11_O_6_^−^	287.0561	151	3.2	2.0	0.8	0.4	0.3	0.5
Eriodictyol isomer	2.87	287.0568	C_15_H_11_O_6_^−^	287.0561	151	0.9	1.5	0.3	0.3	0.1	0.3
Dicaffeoyl-quinic acid IV	3.22	515.1184	C_25_H_23_O_12_^−^	515.1195	353, 173, 179	0.1	0.1	0.4	0.4	0.1	0.1
Tricaffeoyl-quinic acid	3.23	677.1498	C_34_H_29_O_15_^−^	677.1512	515, 353, 191, 179, 173	3.1	2.1	8.7	9.6	2.5	1.4
Naringenin *	3.53	271.0605	C_15_H_11_O_5_^−^	271.0612	151, 119	6.0	3.4	3.6	1.7	7.8	2.2
Naringenin calchone *	3.63	271.0605	C_15_H_11_O_5_^−^	271.0612	151, 119	39.4	57.2	26.3	38.5	19.9	23.7
Hesperetin	3.69	301.0707	C_16_H_13_O_6_^−^	301.0718	151	5.9	2.9	4.3	3.2	4.5	4.2

* Confirmed by authentic standards.

## Data Availability

The data presented in this study are available on request from the corresponding authors.

## Supplementary Materials

The following supplementary data can be downloaded at: https://www.mdpi.com/article/10.3390/biom13010117/s1, Figure S1. ^1^H NMR (CD_3_OD, 600 MHz) of naringenin calchone. Figure S2. ^1^H, ^1^H COSY NMR (CD_3_OD, 600 MHz) of naringenin calchone. Figure S3. ^1^H,^13^C-HSQC NMR (CD_3_OD, 600 MHz) of naringenin calchone. Figure S4. ^1^H,^13^C-HMBC NMR (CD_3_OD, 600 MHz) of naringenin calchone. Figure S5. Calibration curves for glutamic acid and aspartic acid. Figure S6. Calibration curves for 5′-AMP and 5′-GMP. Figure S7. ESI-MS/MS spectra of isomer ions at *m/z* 515.1178 putatively identified as dicaffeoyl-quinic acids I-IV. Figure S8. ESI-MS/MS spectrum of the ion at *m/z* 677.1498 putatively identified as tricaffeoyl-quinic acid. Figure S9. ESI-MS/MS spectra of isomer ions at *m/z* 341.0868 putatively identified as caffeic acid hexosides I-III. Table S1. Tomato sample weights and yield of methanolic extracts. Table S2. NMR signal assignments for naringenin calchone.
