# Supplementary material for: Taste Compounds and Polyphenolic Profile of Tomato Varieties Cultivated with Beneficial Microorganisms: A Chemical Investigation on Nutritional Properties and Sensory Qualities
Source: Biomolecules. 2023 Jan 6;13(1):117. doi: 10.3390/biom13010117 (PMC9855395; doi:10.3390/biom13010117)
Supplement: Supplementary file 1 [file biomolecules-13-00117-s001.zip › biomolecules-2100432-supplementary.pdf]

SUPPLEMENTARY MATERIALS

Article

Taste compounds and polyphenolic profile of tomato varieties cultivated with beneficial microorganisms: a chemical investigation on nutritional properties and sensory qualities

Carlo Francesco Morelli[1], Adele Cutignano[2,*], Giovanna Speranza[1], Gennaro Roberto Abbamondi[2], Marco Rabuffetti[1], Carmine Iodice[2], Rocco De Prisco[2] and Giuseppina Tommonaro[2,*]

1 Department of Chemistry, University of Milan, Via Golgi 19, 20133, Milan, Italy
2 National Research Council (CNR), Institute of Biomolecular Chemistry (ICB), Via Campi Flegrei, 34, 80078 Pozzuoli, Italy
* Correspondence: acutignano@icb.cnr.it (AC); gtommonaro@icb.cnr.it (GT); +39 081 8675313 (AC); +39 081 8675029 (GT)

Figure S1. ^{1}H NMR (CD_3OD, 600 MHz) of Naringenin calchone.

Figure S2. ^{1}H, ^{1}H COSY NMR (CD_3OD, 600 MHz) of Naringenin calchone.

Figure S3. ^{1}H,^{13}C-HSQC NMR (CD_3OD, 600 MHz) of Naringenin calchone.

Figure S4. ^{1}H,^{13}C-HMBC NMR (CD_3OD, 600 MHz) of Naringenin calchone.

Figure S5. Calibration curves for glutamic acid and aspartic acid.

Figure S6. Calibration curves for 5′-AMP and 5′-GMP.

Figure S7. ESI-MS/MS spectra of isomer ions at *m/z* 515.1178 putatively identified as dicaffeoyl-quinic acids I-IV.

Figure S8. ESI-MS/MS spectrum of the ion at *m/z* 677.1498 putatively identified as tricaffeoyl-quinic acid.

Figure S9. ESI-MS/MS spectra of isomer ions at *m/z* 341.0868 putatively identified as caffeic acid hexosides I-III.

Table S1. Tomato sample weights and yields of methanolic extracts.

Table S2. NMR signal assignments for Naringenin calchone.

Figure S1. ^{1}H NMR (CD_3OD, 600 MHz) of Naringenin calchone

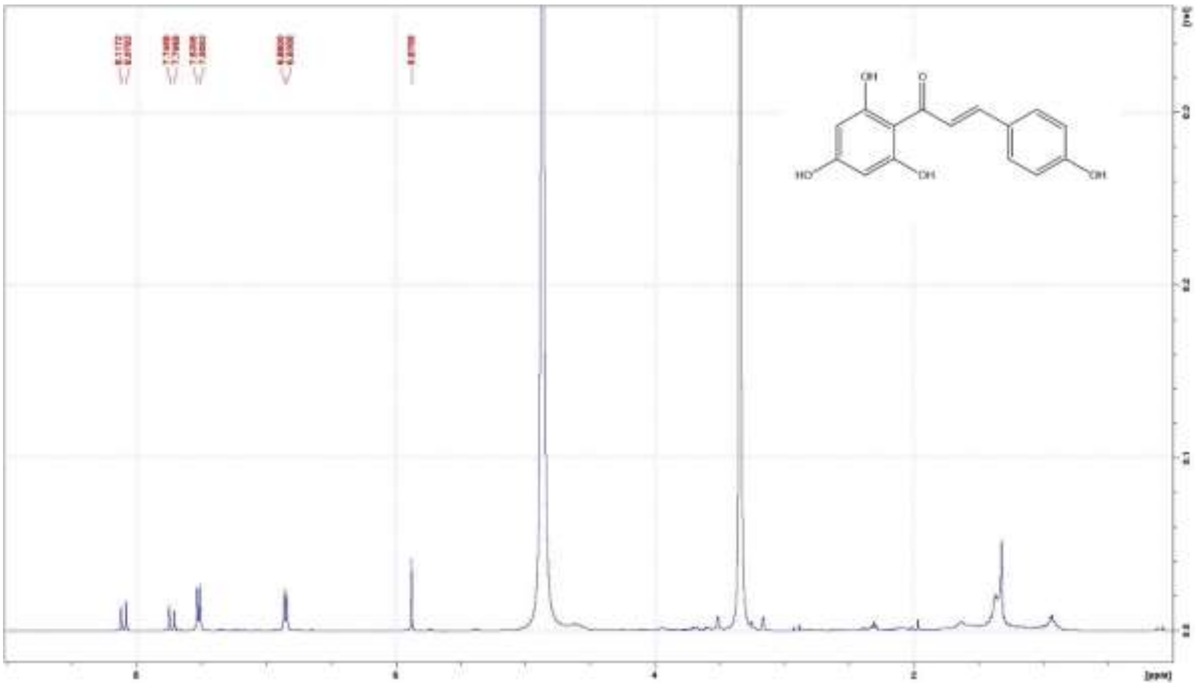

Figure S2. ^{1}H, ^{1}H COSY NMR (CD_3OD, 600 MHz) of Naringenin calchone.

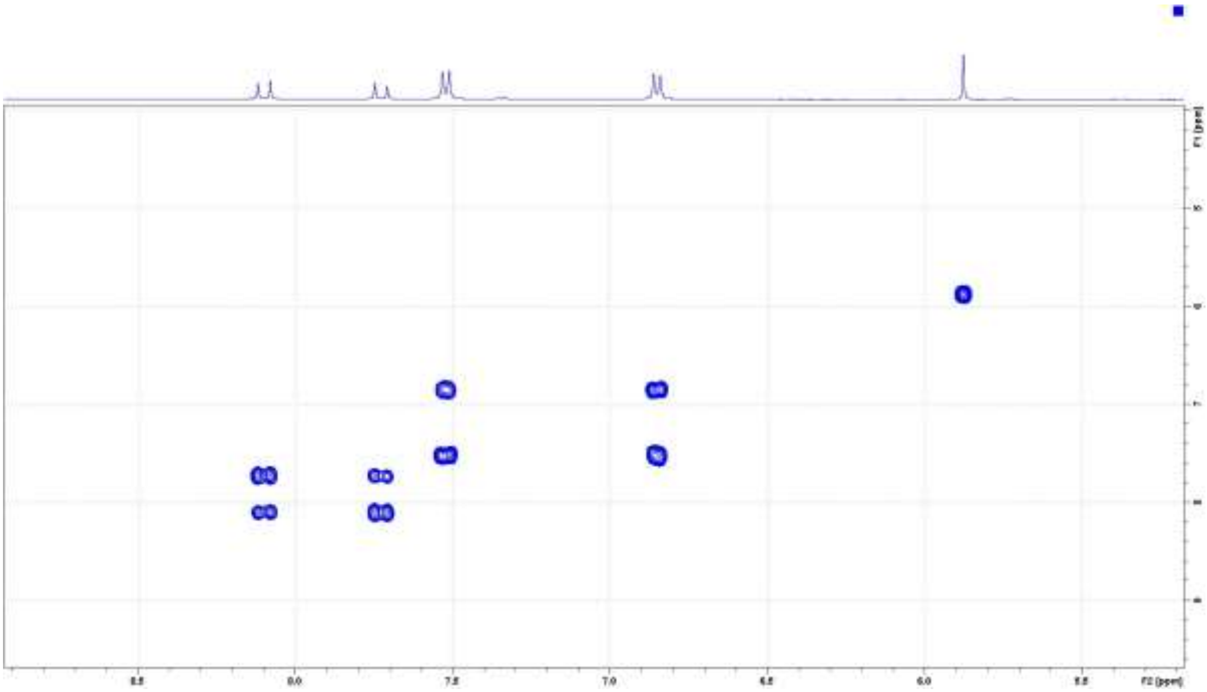

Figure S3. $^{1}H,^{13}C$-HSQC NMR (CD_3OD, 600 MHz) of Naringenin calchone.

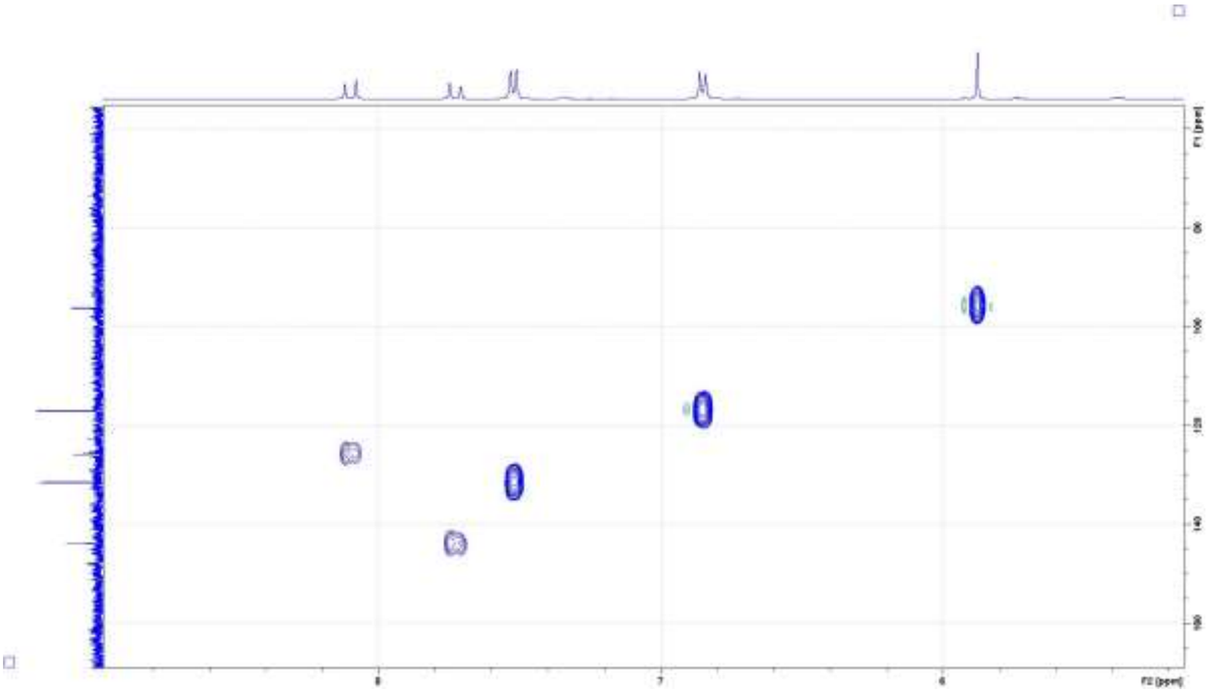

Figure S4. $^{1}H,^{13}C$-HMBC NMR (CD_3OD, 600 MHz) of Naringenin calchone

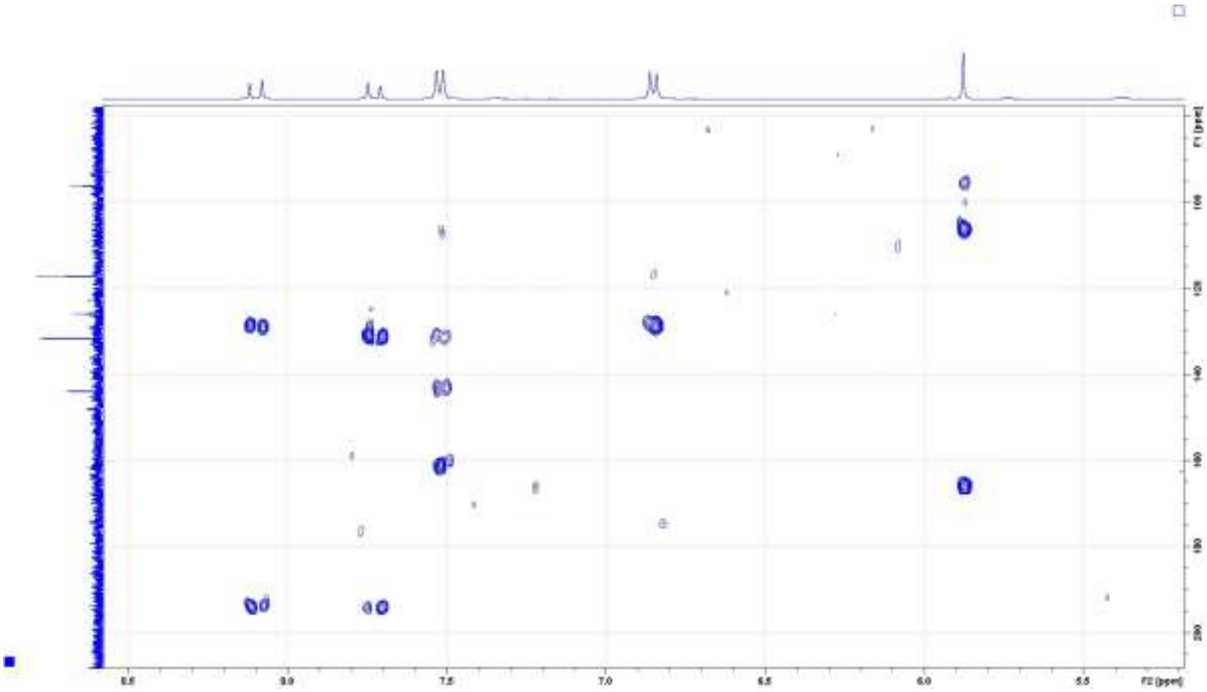

Figure S5. Calibration curves for the quantitative determination of glutamic acid and aspartic acid.

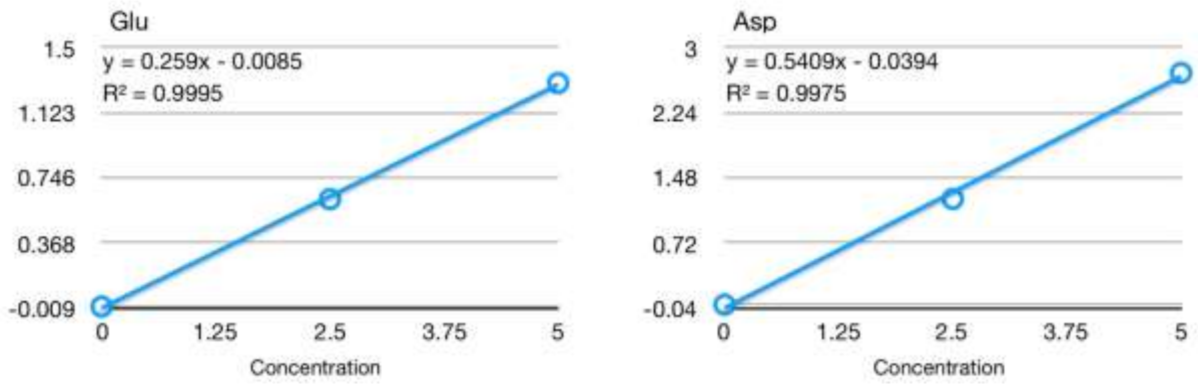

Figure S6. Calibration curves for the quantitative determination of 5′-AMP and 5′-GMP.

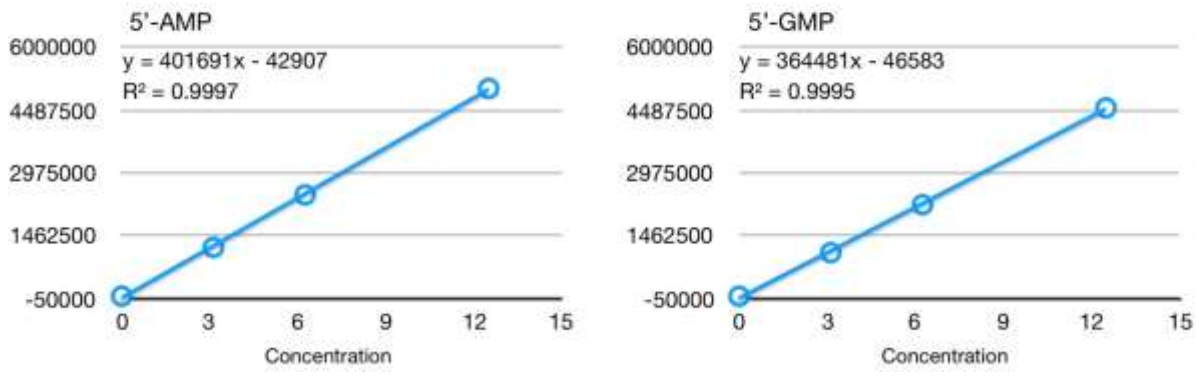

Figure S7. ESI-MS/MS spectra of isomer ions at *m/z* 515.1178 putatively identified as dicaffeoyl-quinic acids I-IV.

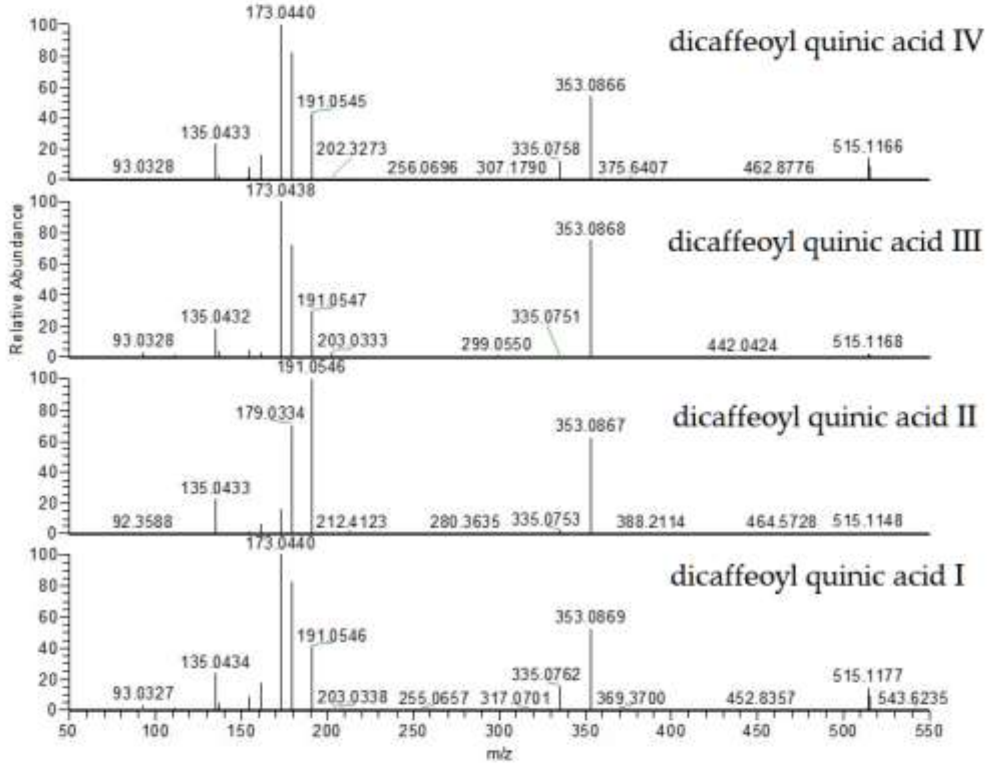

Figure S8. ESI-MS/MS spectrum of the ion at *m/z* 677.1498 putatively identified as tricaffeoyl-quinic acid.

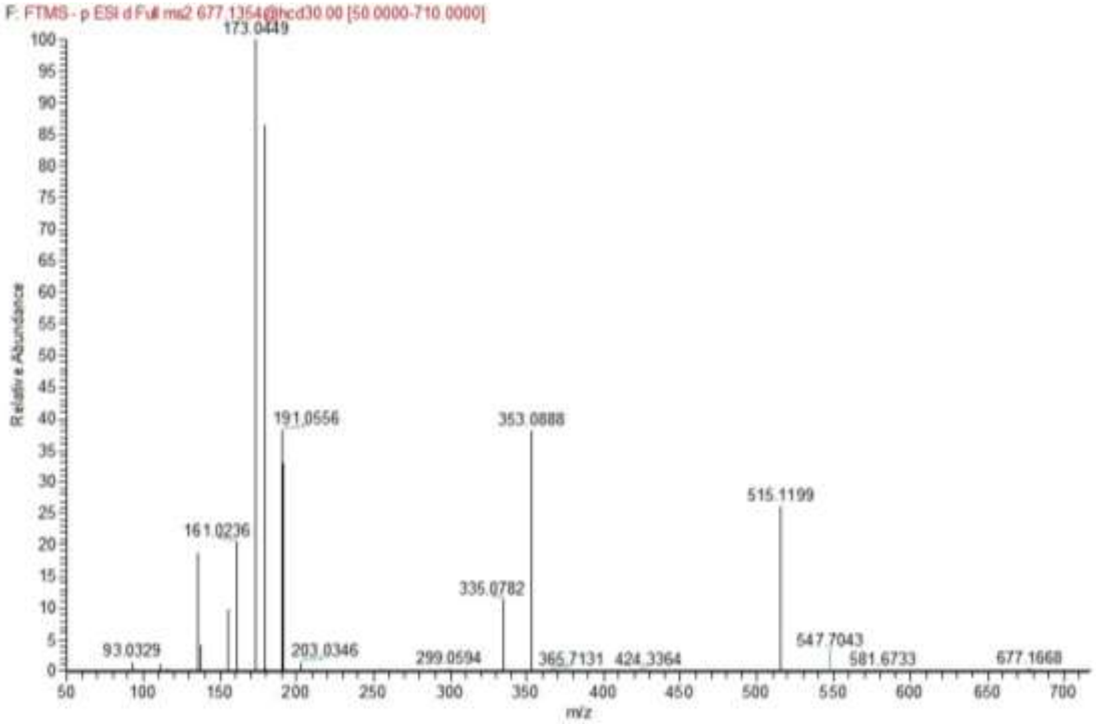

Figure S9. ESI-MS/MS spectra of isomer ions at *m/z* 341.0868 putatively identified as caffeic acid hexosides I-III.

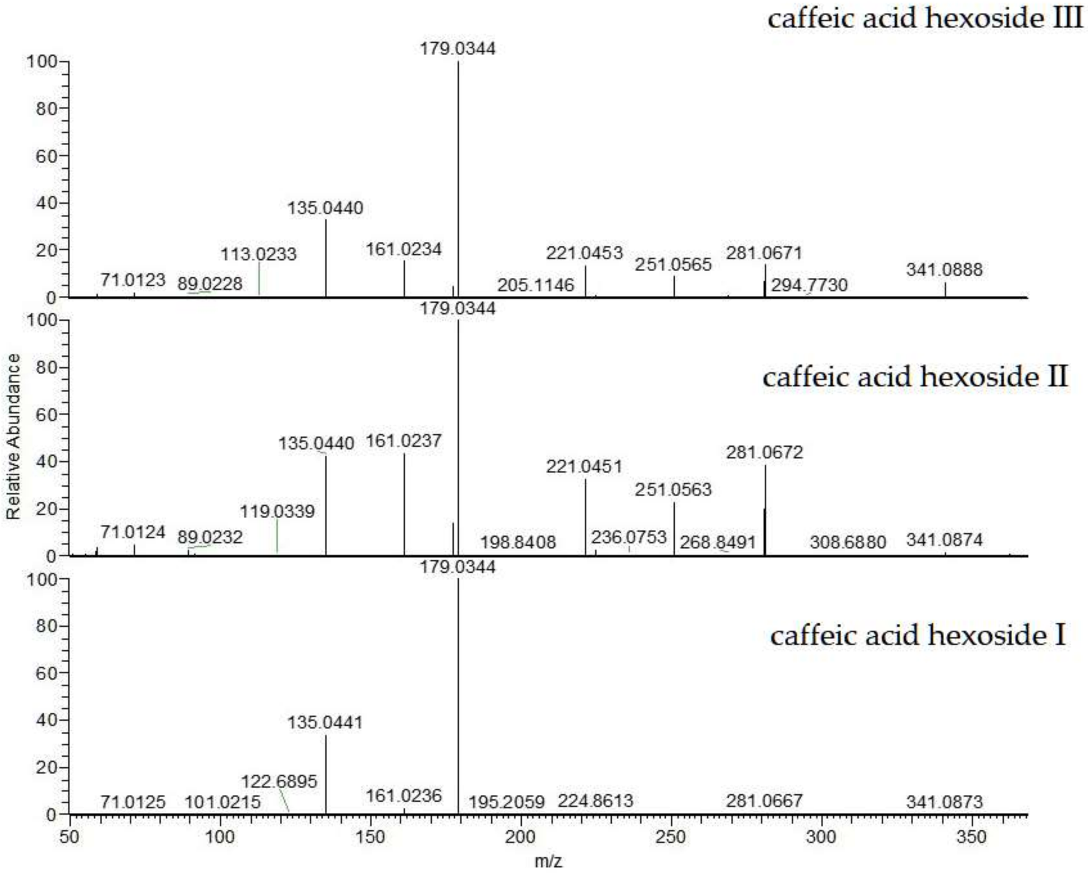

Table S1. Tomato sample weights and yields of methanolic extracts.

**Samples**	**Tomato weight (g)**	**Methanolic extract (mg)**
**SMC3**	100	750.5
**SMC3 + EM**	182	813.2
**CO**	128	640.5
**CO + EM**	120	609.4
**BW**	140	471.5
**BW + EM**	100	253.3

Table S2. NMR signal assignments for Naringenin calchone.

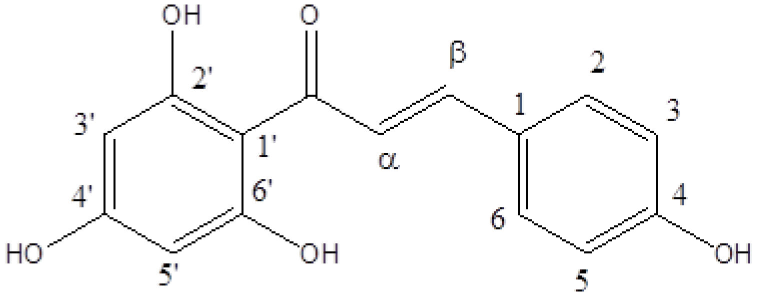

Position	^{1}H, δ, m (J, Hz)	^{13}C, ppm
1	-	128.3
2, 6	7.52 d (8.6)	131.4
3, 5	6.85, d (8.6)	116.7
4	-	160.8
α	8.10, d (15.6)	125.7
β	7.73, d (15.6)	144.0

1′	-	106.1
2′, 4′, 6′	-	165.8
3′, 5′	5.88, s	95.7
CO	-	193.6
